# Relationship between point-of-care ultrasound venous congestion assessment parameters, intravenous pressure, and venous return: a post-hoc analysis of a prospective cohort study

**DOI:** 10.1186/s13089-025-00421-9

**Published:** 2025-02-27

**Authors:** Martin Ruste, Quentin Delas, Rehane Reskot, Jean-Luc Fellahi, Matthias Jacquet-Lagrèze

**Affiliations:** 1 Service d’anesthésie-réanimation, Hôpital Louis Pradel, Hospices Civils de Lyon, Bron, France; 2 Faculté de médecine Lyon Est, Université Claude Bernard Lyon 1, Lyon, France; 3 Laboratoire CarMeN, Inserm UMR 1060, Université Claude Bernard Lyon 1, Lyon, France

**Keywords:** Systemic venous congestion, Ultrasound, Portal pulsatility index, Mean systemic filling pressure, Venous return, VExUS score

## Abstract

**Background:**

A recent study suggested that point-of-care ultrasound (POCUS) venous congestion assessment poorly describes the changes in venous return during a fluid challenge. The aim of the present study was to explore the relationship between POCUS venous congestion assessment parameters and the determinants of venous return in steady state and during a fluid challenge.

**Methods:**

This study is a post-hoc analysis of a single-centre prospective cohort study of patients presenting acute circulatory failure and venous congestion. The protocol consisted in a fluid administration of 4mL/kg over five minutes, just preceded and followed by the acquisition of haemodynamic data and POCUS venous congestion assessment parameters (VExUS score and portal pulsatility index, PPi). Venous return (dVR) was defined as the difference between mean systemic filling pressure analogue estimated by the mathematical approach of Parkin and Leaning (Pmsa) and central venous pressure (CVP). Relationships between Pmsa, CVP, dVR, and VExUS score and PPi were analysed using linear regression and Jonckheere-Terpstra test for trend.

**Results:**

Thirty-two patients were included in the analysis. Fluid challenge induced a significant increase in CVP, Pmsa, dVR, and VExUS score. In steady state, there was a significant association of VExUS score and PPi with CVP (*P-*value = 0.006 and 0.002, respectively) and Pmsa (*P-*value = 0.004 and 0.003, respectively) but not with dVR (*P-*value = 0.943 and 0.408, respectively). The variations induced by fluid challenge in CVP, Pmsa and dVR were not associated with variations in PPi (*P-*value = 0.844, 0.912 and 0.716, respectively). Patients without VExUS score increase during the fluid challenge presented a higher increase in Pmsa than patients with an increase in VExUS score.

**Conclusion:**

In steady state, POCUS venous congestion assessment parameters are associated with CVP and Pmsa but not with dVR. After fluid administration, changes in POCUS venous congestion assessment parameters were not associated with changes in CVP, Pmsa, and dVR.

## Background

Point-of-care ultrasound (POCUS) venous congestion assessment has gained attention in recent years in critically ill, nephrological, and cardiological patients, as a marker of a “reduced venous compliance state” [1]. It is most commonly assessed by measurement of the inferior vena cava diameter and Doppler assessment of the portal, intra-renal, and hepatic veins. If one can consider it as a maker of intravascular fluid volume or cardiac function status, experts advise to better consider it as a fluid tolerance marker [2, 3]. Venous congestion assessment has indeed been suggested to reflect the relationship between the right arterial pressure and the mean systemic filling pressure, which are mediated by the cardiac function, the intravascular volume, and the vessels compliance property [3]. Our group has previously shown that the changes in cardiac index poorly describe the response of the portal pulsatility index (PPi) and the Venous Excess UltraSound (VExUS) score to a fluid challenge in patients with acute circulatory failure and venous congestion [4]. To describe venous return, Guyton et al. initially used a model assessing the pressure gradient for venous return (dVR), defined as the difference between the mean systemic filling pressure and the right atrial pressure [5]. Mean systemic filling pressure can be estimated from the cardiac output (CO), the mean arterial pressure (MAP), and the central venous pressure (CVP) using a mathematical model developed by Parkin and Leaning [6]. The present post-hoc analysis was carried out on our previous prospective cohort of patients in whom CO, MAP, and CVP were monitored [4] to explore the relationship between POCUS venous congestion assessment parameters and dVR, as well as its determinants, at steady state and during a fluid challenge.

## Methods

### Study design and participants

The study is a post-hoc analysis of a single-centre prospective cohort study (*Hôpital Louis Pradel*, Cardiovascular and thoracic centre, *Hospices Civils de Lyon*, Bron, France). Patients were included if they presented acute circulatory failure (defined by the need for vasopressor and at least one criteria of impaired peripheral perfusion) and had an inferior vena cava maximum diameter > 20 mm. The exact criteria for eligibility were fully described in the initial study [4]. For the present analysis, patients with missing values in CO, MAP and CVP were also excluded as these variables are required to assess the mean systemic filling pressure.

### Objectives and definitions

The primary objective was to describe the relationship between POCUS venous congestion assessment parameters and the dVR. The latter was defined as the difference between the mean systemic filling pressure analogue (estimated by the mathematical model, Pmsa) and the CVP. The secondary objective was to describe this relationship during a fluid challenge.

Pmsa was assessed as described by Parkin and Leaning [6] according to the following formulas:

$${\text{Pmsa}} = 0.96\left( {{\text{CVP}}} \right) + 0.04\left( {{\text{MAP}}} \right) + {\text{c}}\left( {{\text{CO}}} \right)$$

Where c was determined by [7]:

$$c = \frac{{0.96 \times 0.038\left( {94.17 + 0.193 \times {\text{age}}} \right)}}{{4.5\left( {{{0.99}^{{\text{age}} - 15}}} \right) \times 0.007184\left( {{\text{heigh}}{{\text{t}}^{0.725}}} \right)\left( {{\text{weigh}}{{\text{t}}^{0.425}}} \right)}}$$

The dVR and the resistance to venous return (RVR) were then calculated using the formulas [5]:

$${\text{dVR}} = {\text{Pmsa}} - {\text{CVP}}$$

$${\text{RVR}} = \frac{{{\text{dVR}}}}{{{\text{CO}}}}$$

### Protocol and data handling

The protocol consisted in a fluid administration of 4mL/kg of balanced crystalloid over five minutes (lactated Ringer’s solution at room temperature), just preceded and followed by the acquisition of haemodynamic data and POCUS venous congestion assessment parameters. These included invasive measurement of arterial pressure with CO estimation by pulse contour analysis using FloTrac™ transducer / Hemosphere™ monitor (Edwards Lifesciences, Irvine, CA, USA) or PiCCO^®^ transducer and monitor (Pulsion Medical Systems, Munich, Germany); CVP estimation within the venous central line; POCUS assessment of systemic venous congestion with quantification of PPi [8] and calculation of VExUS score [9]; and peripheral perfusion assessment with standardised capillary refill time [10] and peripheral perfusion index using Intellivue MX750 monitor (Philips Healthcare, Andover, MA, USA). There were no changes in mechanical ventilation parameters and catecholamine administration (if any) between the two timings of evaluation. Variables collected at baseline and data acquisition were extensively described in the initial study [4].

### Statistical analysis

Data were expressed as mean ± standard deviation (SD), median [25th − 75th percentile], or count and percentage, as appropriate. No imputation was carried out for missing values. Paired Student t test and Wilcoxon signed-ranked test were used to describe haemodynamic variations observed during the fluid challenge. To analyse the relationship in steady state of Pmsa, CVP, and dVR with POCUS systemic venous congestion assessment, we grouped the values obtained before and after the fluid challenge in one set of measurement. Then, the Jonckheere-Terpstra test for trend was used for VExUS score and linear regression for PPi. To assess the dynamic relationship during the fluid challenge of the precited parameters with PPi, linear regression between the absolute variations observed were carried out. For VExUS score we categorized dynamic changes observed during fluid challenge as follows: (1) VExUS score 1–2 at baseline with no VExUS score variation after the fluid challenge; (2) VExUS score 1–2 at baseline with increase in VExUS score after the fluid challenge; (3) VExUS score 3 at baseline. We then compared the variations in Pmsa, CVP, and dVR in these three groups using a Kruskal-Wallis rank sum test. The statistical analysis was performed using R version 4.3.2 (R Core Team, 2017, Vienna, Austria). All the tests were two-sided and a *P*-value < 0.05 was considered significant.

## Results

**Fig. 1:**
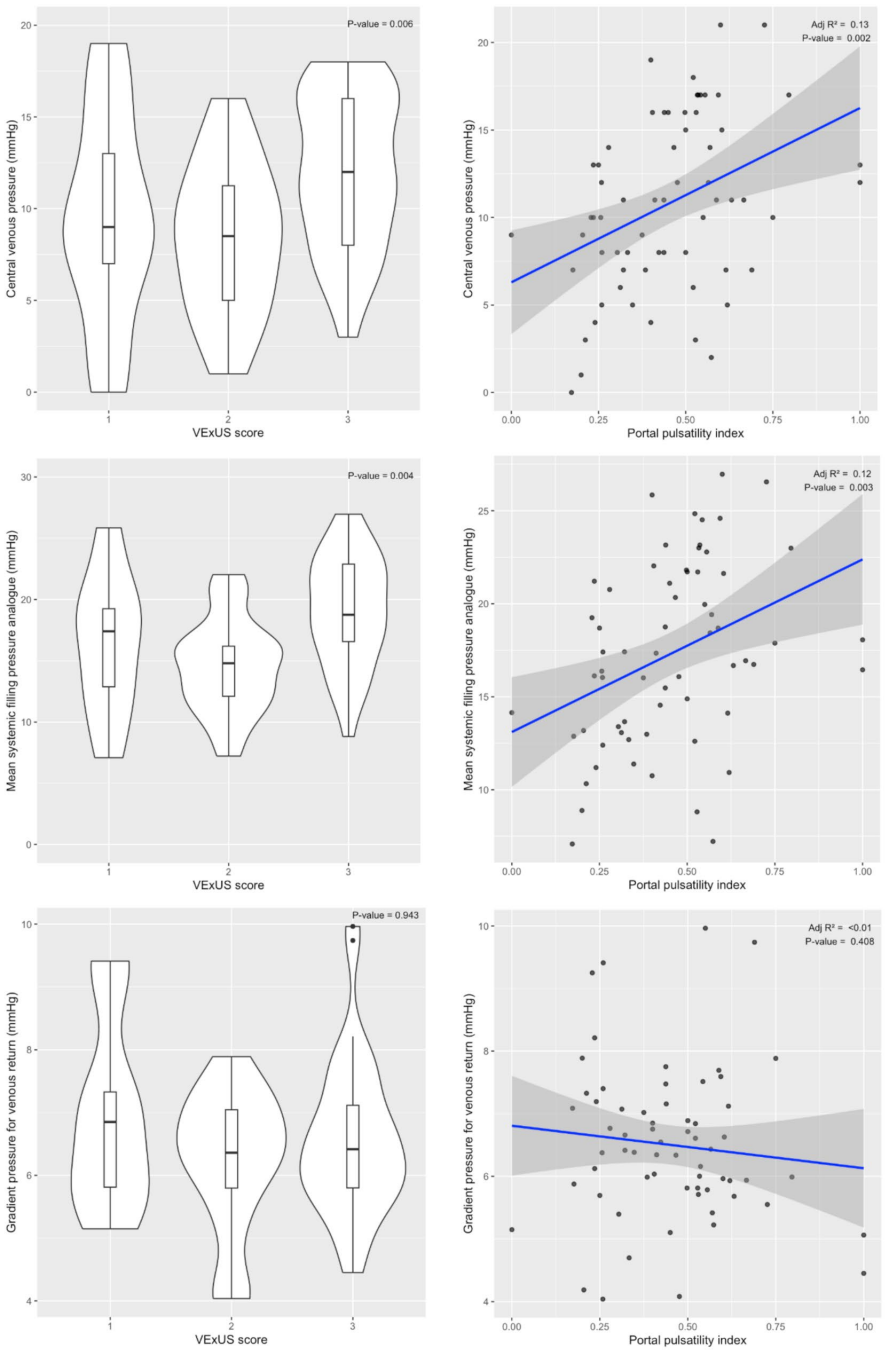
Relationship of central venous pressure, mean systemic filling pressure analogue, and gradient pressure for venous return with VExUS score and portal pulsatility index Footnote: Left column: violin and boxplot representing the relationship of VExUS score with central venous pressure, mean systemic filling pressure analogue, and gradient pressure for venous return. *P*-value is for Jonckheere-Terpstra test. Right column: linear regression of portal pulsatility index and central venous pressure, mean systemic filling pressure analogue, and gradient pressure for venous return. The mean systemic filling pressure analogue was determined by the mathematical model of Parkin and Leaning [6]. The gradient pressure for venous return was defined as the difference between the mean systemic filling pressure analogue and the central venous pressure

Thirty-two patients (90% of the initial study population) were included in the analysis (enrolment between 10 July 2023 and 03 June 2024). The main characteristics at baseline of the study population are reported in Table 1. Fluid challenge induced a significant increase in Pmsa, CVP, dVR, and VExUS score (Table 2). In steady state, we observed a significant increase of VExUS score with that of Pmsa and CVP but not with dVR. There was a weak correlation between PPi and Pmsa, and between PPi and CVP, but not between PPi and dVR (Fig. 1). During the fluid challenge, there was no significant relationship between the changes in PPi and the changes in Pmsa (adjusted R^2^ -0.03, *P-*value 0.912), CVP (adjusted R^2^ -0.03, *P-*value 0.844), and dVR (adjusted R^2^ -0.03, *P-*value 0.716). Thirteen patients had a VExUS score grade 3 at baseline. Among the other patients, none presented a decrease and 10 patients presented an increase in VExUS score. The changes in Pmsa, CVP, dVR, and other haemodynamic parameters during the fluid challenge and according to changes in VExUS score are reported in Table 3.

**Table 1:** Population characteristics at baseline

Variable	*N* = 32
Age, years	70 [62–77]
Height, cm	172 ± 9
Sex, female, *n* (%)	10 (31)
Body Mass Index, kg/m^2^	25 ± 4
Admission category, *n* (%)	
Cardiac surgery	27 (84)
Other surgery	1 (3)
Medical	4 (13)
SOFA score	8 ± 3
Left ventricular ejection fraction, %	44 ± 8
Mechanical ventilation, yes, *n* (%)	19 (60)
Renal replacement therapy, yes, *n* (%)	3 (10)
Right ventricular dysfunction, *n* (%) *	
Yes	20 (63)
No	4 (13)
Missing	8 (25)
Fluid responsiveness, yes, *n* (%)	13 (41)
Arterial lactate, mmol/L	2.7 [2.2–3.3]
Norepinephrine equivalent, µg/kg/min **	0.20 [0.09–0.45]
Vasoactive inotropic score***	22 [10–53]

Values are expressed as median [25th − 75th percentile], mean ± standard deviation, or count (%). SOFA: Sepsis Organ Failure Assessment [16]; *Defined by the presence of at least one of the following parameters: right ventricular fractional area change < 35%, tricuspid annular plane exclusion < 16 mm, or tricuspid annular systolic excursion velocity < 10 cm/s; ** As described by Kotani et al. [17]; *** As described by Koponen et al. [18]

**Table 2:** Haemodynamic variations observed during the fluid challenge

Variable	*n* = 32		*P*-value
	Before fluid challenge	After fluid challenge	
Cardiac index, L/min/m^2^	2.6 ± 0.7	2.8 ± 0.7	0.005
Mean arterial pressure, mmHg	67 ± 7	72 ± 6	< 0.001
Central venous pressure, mmHg	10 ± 5	12 ± 5	< 0.001
Mean systemic filling pressure analogue, mmHg	16 ± 5	19 ± 5	< 0.001
Gradient pressure for venous return, mmHg	6 ± 1	7 ± 1	< 0.001
Resistance to venous return,	1.3 ± 0.3	1.3 ± 0.3	0.599
Portal pulsatility index, %	42 ± 0.18	48 ± 0.20	0.134
VExUS score			0.003
1	6 (19)	3 (9)	
2	13 (41)	7 (23)	
3	13 (41)	22 (69)	
Capillary refill time, s	4.4 [3.2–9.9]	4.5 [3.2–7.5]	0.162
Peripheral perfusion index	0.97 [0.34–1.70]	1.10 [0.35–1.50]	0.648

Values are expressed as median [25th − 75th percentile], mean ± standard deviation, or count (%). *P*-value are for paired Student t test or Wilcoxon signed-ranked test (VExUS score)

**Table 3:** Haemodynamic variations observed during the fluid challenge according to changes in vexus score

Variables	VExUS score 1–2 at baseline		VExUS score 3 at baseline	*P*-value
	No change in VExUS score (*n* = 9)	VExUS score increase (*n* = 10)	(*n* = 13)	
Before the fluid challenge				
Cardiac index, L/min/m^2^	2.1 [2.1–2.7]	2.6 [2.5–2.9]	2.5 [2.0-3.3]	0.536
Mean arterial pressure, mmHg	67 [62–71]	68 [64–71]	68 [63–72]	0.909
Central venous pressure, mmHg	8 [4–9]	12 [8–16]	11 [8–13]	0.034
Mean systemic filling pressure analogue, mmHg	13 [11–14]	17 [16–22]	17 [15–19]	0.029
Gradient pressure for venous return, mmHg	6 [5–7]	6 [6–7]	6 [6–7]	0.931
Resistance to venous return,	1.3 [1.3–1.5]	1.2 [1.1–1.5]	1.3 [1.2–1.4]	0.317
Fluid responsiveness, yes, *n* (%)	5 (56)	2 (20)	6 (46)	0.252
VExUS score				NA
1	3 (33)	3 (30)	0 (0)	
2	6 (67)	7 (70)	0 (0)	
3	0 (0)	0 (0)	13 (100)	
Portal pulsatility index, %	25 [20–30]	41 [40–46]	53 [44–57]	0.004
Capillary refill time, s	6.1 [2.9–10.6]	4.9 [4.0-7.8]	4.2 [3.3–4.5]	0.807
Peripheral perfusion index	0.80 [0.33–1.10]	1.50 [1.00-2.97]	0.80 [0.20–1.63]	0.102
Relative variations during the fluid challenge				
Cardiac index, %	12 [0–14]	0 [-7- 7]	9 [1–15]	0.160
Mean arterial pressure, %	9 [0–13]	8 [-2- 13]	6 [2–10]	0.787
Central venous pressure, %	33 [25–44]	8 [2–34]	31 [21–42]	0.085
Mean systemic filling pressure analogue, %	22 [12–32]	5 [3–21]	20 [17–29]	0.038
Gradient pressure for venous return, %	8 [3–11]	4 [-2- 7]	9 [1–12]	0.450
Resistance to venous return, %	-2 [-7- 0]	2 [-5- 8]	-3 [-7- 0]	0.299
Portal pulsatility index, %	18 [8–23]	28 [12–47]	-4 [-20- 37]	0.259
Capillary refill time, %	-13 [-19- -4]	-6 [-16-19]	1 [-3- 7]	0.235
Peripheral perfusion index, %	9 [-3- 37]	-9 [-32-18]	0 [-16- 44]	0.327

*P*-values are for Kruskal-Wallis rank sum test (continuous variables) and Fisher’s exact test (categorical variables)

## Discussion

The present study confirms that, although VExUS score and PPi are associated with CVP and Pmsa at steady state, they poorly describe the dVR, at both steady state and during a fluid challenge. The relationship between the VExUS score and the CVP observed at steady state confirms previous validation studies of the score [11, 12] and is physiologically consistent with the Guyton model that considers CVP as a backward pressure to venous return. Nevertheless, this association remains weak, with important overlap in CVP values between the different VExUS grades, limiting the use of the VExUS score for the estimation of right atrial pressure, except possibly for the prediction of high right atrial pressure [11]. At steady state, a similar association was observed herein between the POCUS venous congestion assessment parameters and Pmsa. These results are physiologically relevant as Pmsa represents the intravenous pressure. Both POCUS venous congestion assessment parameters and Pmsa have previously been described as reflecting “organ afterload” and associated with acute kidney injury [9, 13]. However, we found no significant association of POCUS venous congestion assessment parameters with dVR, which confirms that POCUS venous congestion is not a marker of venous return in steady state, and must be strictly interpreted as a marker of venous distention.

It has been shown that fluid administration increases the mean systemic filling pressure to the same magnitude regardless of the degree of fluid responsiveness, but dVR increases only in fluid responders [14]. Based on the hypothesis that POCUS venous congestion parameters can be markers of Pmsa and CVP, the lack of relationship between changes in these parameters during the fluid challenge was unexpected, as was the fact that patients with an increase in VExUS score had a lower increase in Pmsa than patients with no change in VExUS score. Since the Pmsa and CVP before the fluid challenge were lower in the patients without changes in VExUS score, it is possible that the VExUS score can only increase at a high threshold of intravenous pressure, and that an increase in the VExUS score may highlight the inability of the Pmsa to increase further. However, the grading of the score up to 3 limits the interpretability of results in patients with more severe POCUS venous congestion. Furthermore, the use of thresholds in PPi to calculate the VExUS score may have participated in an artificial categorization of the population in the present analysis.

The present study has several limitations. First, it is a post-hoc analysis of a small single-centre cohort in a specific population, which limits the generalizability of the results. Second, the mathematical analysis used to assess dVR determinants is susceptible to collinearity between the variables. Third, CVP is only a surrogate marker of right atrial pressure. Nevertheless, the present study is the first, to the best of our knowledge, to rigorously study the relationship between POCUS congestion assessment and haemodynamic determinants for venous return. It confirms that POCUS congestion assessment parameters are associated with intravenous pressure at steady state but not with venous return. It also highlights several limitations, questioning the ability of POCUS venous congestion assessment for measuring changes in intravenous pressure induced by a fluid administration. The assessment of fluid-induced changes in both haemodynamic and POCUS venous congestion parameters could be predicted by the passive leg raising test [15]. Whether the combination of these different markers of venous congestion could be useful in assessing fluid tolerance remains to be investigated.

## Data Availability

All de-identified datasets may be available for secondary analysis upon reasonable request to the corresponding author.

## Abbreviations

CO: Cardiac output
CVP: Central venous pressure
dVR: Pressure gradient for venous return
ICU: Intensive care unit
MAP: Mean arterial pressure
Pmsa: Mean systemic filling pressure analogue
PPi: Portal pulsatility index
RVR: Resistance to venous return
SOFA: Sepsis organ failure assessment
VExUS: Venous excess ultrasound grading system
